# Memoirs of the Royal Academy of Sciences of Sweden for the Year 1778

**Published:** 1782

**Authors:** 


III. *Kongl. Vetenskaps Academiens Handlingar för År.* 1778, i. e. *Memoirs of the Royal Academy of Sciences of Sweden for the year* 1778. 8vo. Stockholm.

I. *OBSERVATIONS on the climate of Sweden with regard to heat and cold, by* Peter Wargentin, *Secretary to the Academy, &c.* This paper is the result of twenty years observations, and is intended as a supplement to a former paper on the same subject, which our author communicated to the Academy twenty years ago. The two papers include a series of 39 years. The observations were made with Celsius's thermometer, which is but little known out of Sweden; in in quoting them we ſhall therefore reduce them to Fahrenheit's ſcale, which we could wiſh to ſee univerſally adopted by all the learned in Europe, as the different thermometers of de Liſle, de Reaumur, &c. which are at preſent in uſe in different countries, ſerve only to confuſe the philoſophical reader.

It ſeems that in Sweden the weather is not conſidered as cold till the mercury has fallen five degrees below 0. In the courſe of 39 years the coldeſt day was the 7th of January 1760, when the thermometer ſunk 20 degrees below 0.

In the ſummer of 1775, which was remarkable for its warmth, the mercury roſe 41 times to 75°, and 9 times to 87°.

Our author obſerves that the changes in the temperature of the atmoſphere are very ſudden. The mean height of the thermometer in Sweden is ſaid to be about 42°.

II. *An account of the Bezoar generated in the ſtomach of horſes in Japan, by* C. P. Thunberg, M. D.—This ſubſtance, which when broken, appears to be compoſed of different ſtrata, without any nucleus, is met with of different ſizes. The phyſician to the emperor of Japan made a preſent to our author of one which weighed two pounds and ſix ounces.

III.

III. *An account of four cafes of hydrocele cured by cauftic, by* Sir Olaus Acrel, Knt. M. D. &c. This paper is communicated to the academy by Dr. Gahn, who has added a fifth cafe of the fame kind from his own obfervation, together with fome general remarks on the treatment of hydroceles, in which he gives the preference to a fmall cauftic applied after Mr. Elfe's method.

IV. *A remarkable cafe, by* A. Sparrman, M. D. —We have here an account of a patient, who after taking Madame Nouffer's remedy (the fern root) for a fuppofed tænia, voided a confiderable quantity of *nymphæ* of the *mufca meteorica.* Our author fuppofes that the eggs of thefe infects were brought into the body through the anus, in the fame manner as thofe of the *œftrus hæmorrhoidalis* are known to be introduced into the inteftinal canal of horfes.

V. *A cafe of Catalepfy, by* M. Hiontzberg.— The fubject of this cafe was a man of a melancholic temperament, and the difeafe was accompanied with a locked jaw; clyfters, venæfections, warm bathing, finapifms, and a variety of other remedies were prefcribed, and on the fourth day the patient began to recover the faculty of fwallowing. A dofe of fal amarus was then exhibited, and and the patient had ſeveral fœtid mucous ſtools which ſeemed to haſten his recovery.

The ſame writer communicates an account of an oſſification in the cavity of the aorta near the left ventricle of the heart, which produced violent palpitation and difficulty of breathing.

VI. *Caſe of a woman who was bit by a viper*, by M. Hofberg.—The patient was bit in the arm, and the ſymptoms excited were a ſwelling of the limb, heat, ſhivering, ſickneſs at the ſtomach, anxiety, and difficulty of breathing. Our author, who was called to her half an hour after the accident, directed the whole arm to be frequently bathed with olive oil, and a table ſpoonful of the ſame oil to be given internally, at firſt, every half hour, and afterwards every hour, till ſhe vomited. On the ſecond day the patient ſweated profuſely, and the ſwelling and other ſymptoms diſappeared. The author informs us that the efficacy of olive oil has been repeatedly experienced in Sweden, as an antidote to the poiſon of the viper.

Sir Olaus Acrel has added ſome remarks by way of ſupplement to this paper. He recommends, in caſes of this ſort, a treatment ſimilar to that which has been of late adviſed againſt the bite of a mad dog, and which conſiſts in promoting a difcharge from the wound by means of cantharides, mezereum, or fome other irritating fubftance. Two cafes are quoted as proofs of the efficacy of this practice.

VII. *Of the efficacy of arfenic in cancers, by* Mr. Ronnou.—This writer affures us that in the courfe of fifty years, during which he has adminiftered this remedy, he has cured twenty cancerous patients; and he goes fo far as to contend that arfenic is as fpecific in cafes of this kind as mercury is in fyphilis. He gives it internally in folution, in very fmall dofes, after the manner of Le Febre, and in cafes of open cancer applies it at the fame time externally to the ulcer. He obferves, that many years ago at Paris, he faw M. St. Yves cure fmall cancers of the eyelids by a water compofed of elder flower water, quick-lime, and white arfenic.

VIII. *An account of a fingular tumour of the head, by* M. Taxe.—This tumour was on the right fide of the forehead of a girl four years old. It was large, attended with pulfation, and when preffed occafioned *tinnitus aurium* and drowfinefs. Our author fuppofes it to have been a *hernia cerebri*. The paper is accompanied with an engraving of the tumour, and fome remarks are added to it by profeffor Martin, which are intended tended to prove that injuries done to the os frontis are lefs fatal than when they happen to other parts of the head.

IX. *Cafe of a gun-fhot wound, by* J. L. Odhelius, M. D.—In this cafe the wound was inflicted over the left eye-brow. The part healed, and the patient feemed to be doing well, when he was fuddenly feized with convulfions and ftupor, which ended in death. On diffection part of a bullet was found lying on the integuments of the brain.

X. *An account of fome ftatical experiments, by* R. Martin, M. D. &c.—The author himfelf was the fubject of thefe experiments. He has found that the effect of opium is firft to leffen the fenfible heat of the body, and afterwards to promote perfpiration.

XI. *Cafe of a patient who had a cataract in each eye, by* J. L. Odhelius, M. D.—In this cafe both pupils were entirely contracted, fo that the author was obliged to make an artificial pupil, through which he extracted the difeafed lens. Sir O. Acrel, in another paper on the fame fubject, advifes us in cafes of this kind, where it is neceffary to make an artificial pupil, to divide the longitudinal fibres of the iris tranfverfely.

The other papers contained in this volume are a defcription of the *Hudfonia ericoides* LINN. by profeffor Bergius;—A defcription of the *Erica Sparmanni* (an African fhrub fo named in honour of its difcoverer) by profeffor Linnæus;—Remarks on the effects of cold on certain trees and plants; on the degrees of heat at which many plants flowered in Sweden in 1777; a defcription of the *mufca pumilionis*; of the means of deftroying the worm which is fo deftructive to rye; and obfervations on the damage done by the *phalæna tritici* to oats, wheat, and rye, in five feparate papers, by Mr. Bierkander;—an account of the preparation of *mercurius dulcis via humida* (fee vol. II. of our Journal, p. 53); a new method of preparing the *pulvis algerothi* (of this we mean to fpeak more fully in a future number); an account of a new green colour (fee our fecond vol. page 132); experiments with black lead (which prove that it confifts of a peculiar acid, different from all other acids, and which for this reafon the author names *acidum molybdænæ*) all thefe by Mr. Scheele;—a differtation on the genus of *Yerbua*, and particularly of the *Yerbua Capenfis*, by J. R. Forfter, L.L.D. &c;—a defcription of the *Sturnus Dauricus* and *Alanda Mongolienfis*, by profeffor Pallas;—an account of the properties and ufe of the hiccory or or American walnut-tree, by profeſſor Kalm;—experiments relative to the preſence of zinc in iron ore, by Mr. Hielm;—a deſcription of two ores of zinc, by Mr. Brumich;—additions to the natural hiſtory of the rhinoceros, and hippopotamus, by A. Sparrman, M. D.

